# Toripalimab plus chemotherapy for metastatic muscle-invasive bladder cancer with a high tumor proportion score: a case report

**DOI:** 10.3389/fimmu.2024.1485744

**Published:** 2024-12-18

**Authors:** Wei Ning, Pengkang Chang, Ji Zheng, Wei Chen

**Affiliations:** Department of Urology, Urologic Surgery Center, Xinqiao Hospital, Third Military Medical University (Army Medical University), Chongqing, China

**Keywords:** toripalimab, chemotherapy, metastatic muscle-invasive bladder cancer, tumor proportion score, programmed death ligand 1

## Abstract

**Background:**

Radical cystectomy (RC) combined with pelvic lymph node dissection (PLND) is the standard treatment for muscle-invasive bladder cancer (MIBC). For metastatic MIBC patients, platinum-based chemotherapy remains the first choice treatment. However, approximately 50% of patients with metastatic MIBC are ineligible for platinum-based adjuvant chemotherapy because of impaired renal function. In programmed death ligand 1 (PD-L1)-positive patients who cannot tolerate platinum-based chemotherapy, immunotherapy is recommended. Thus, a major shift is taking place in the treatment of patients with metastatic MIBC. There is currently much interest in the use of chemotherapy combined with immunotherapy and maintenance immunotherapy for the treatment of metastatic MIBC.

**Case presentation:**

One patient with metastatic MIBC exhibited promising progression-free survival (PFS) and safety and had good renal function after RC and toripalimab combined with chemotherapy plus toripalimab maintenance therapy.

**Conclusion:**

RC plus adjuvant therapy (toripalimab combined with chemotherapy) plus toripalimab maintenance therapy is a potential treatment option for metastatic MIBC patients who want to prolong their life. Moreover, a high tumor proportion score (TPS) of PD-L1 expression as well as CDKN2A and TP53 mutation levels may predict immunotherapy efficacy and patient prognosis.

## Introduction

Bladder cancer (BC) is a common malignancy worldwide, with approximately 500,000 new cases and more than 170,000 deaths annually (1). Muscle-invasive bladder cancer (MIBC) accounts for nearly 20% of newly diagnosed cases of BC. Despite promising initial results with radical cystectomy (RC) plus pelvic lymph node dissection (PLND), more than half of patients ultimately develop distant metastasis due to disseminated micrometastases. In addition, ileal urinary diversions and ureterocutaneostomy may affect the quality of life (QoL), urinary and sexual function, body image, and mental, social, and emotional health of patients (2, 3). Therefore, there is an urgent need to develop new therapeutic strategies and comprehensive systematic therapies to preserve the bladder and reduce the recurrence rates in this population.

Over the past 4 decades, systematic therapy for MIBC and advanced BC has focused mainly on platinum-based chemotherapy (4). However, the benefit of platinum-based chemotherapy is relatively modest, with a 5–6% overall survival (OS) benefit from neoadjuvant chemotherapy after 10 years and no benefit from adjuvant chemotherapy. Because of the limited OS benefit and significant renal toxicity, platinum-based chemotherapy is underutilized in current clinical practice (5). Recently, the development of sequencing technologies has improved the genomic characterization of BC, which has increased our understanding of the pathogenesis of BC and identified potential therapeutic targets. Despite the high mutational burden of BC, a subset of MIBC and advanced BC patients treated with immune checkpoint inhibitors (ICIs) still exhibit durable responses. Given the encouraging treatment prospects of ICIs, ICIs have been approved for the treatment of several solid tumors. In particular, accumulating evidence has supported the study of the effects of ICIs on urothelial carcinoma (UC) (6, 7).

The Food and Drug Administration (FDA) has approved five ICIs (pembrolizumab, nivolumab, atezolizumab, avelumab and durvalumab) for the treatment of MIBC patients who experience tumor progression during or following platinum-based chemotherapy. In addition, pembrolizumab and nivolumab have also been approved as first-line treatments for MIBC patients who are ineligible for platinum-based chemotherapy and have high expression of programmed death ligand 1 (PD-L1) (8, 9). From clinical studies conducted in China, ICIs have been observed to have preliminary antitumor effects in UC, Hodgkin’s lymphoma, melanoma, non-small-cell lung cancer, gastric cancer, esophageal squamous cell carcinoma, hepatocellular carcinoma, nasopharyngeal carcinoma, colorectal cancer, and microsatellite instability-high (MSI-H)/deficient mismatch repair (dMMR) solid tumors (10). Moreover, it has been approved for the treatment of UC, classical Hodgkin’s lymphoma, non-small-cell lung cancer, and hepatocellular carcinoma in China (11). Additionally, certain new clinical indications for MSI-H/dMMR solid tumors are currently being explored. Toripalimab (TuoyiTM) is the first domestic anti-programmed death receptor 1 (PD-1) monoclonal antibody in China with completely independent intellectual property rights. It is able to bind to PD-1, efficiently blocking its interaction with PD-L1 (12). Compared with other PD-1/PD-L1 inhibitors used in clinical practice, toripalimab has positive therapeutic effects, acceptable safety profiles, and economic benefits (13). Thus, it may provide a potentially effective weapon against cancer for clinicians and patients, especially for patients with a financial burden.

Here, we report one patient with metastatic MIBC subclassified by gene expression profiling. In parallel, we provide a new treatment strategy consisting of adjuvant therapy (toripalimab, gemcitabine, and cisplatin) after RC plus PLND and maintenance therapy with toripalimab. The patient agreed with the disclosure of his clinical data.

## Case presentation

**Figure 1:**
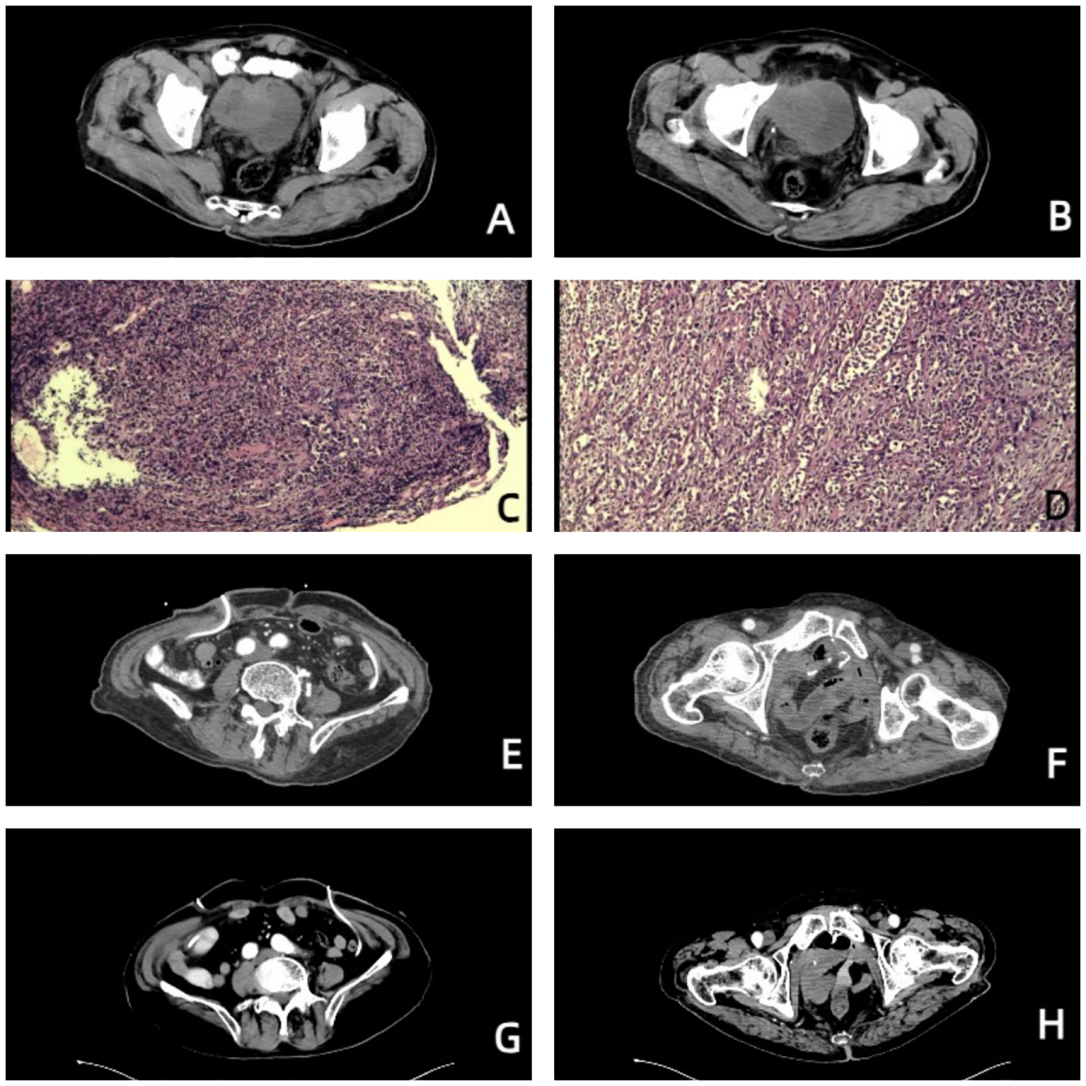
Images of the patient throughout the treatment. **(A, B)** Before treatment, urinary tract CT revealed that the right wall of the bladder was not uniformly thickened, a mass with soft tissue density was visible, the size was approximately 5.5*5.2 cm, and the pelvic lymph nodes were enlarged. **(C)** Pathological diagnosis via cystoscopy combined with imaging, morphology, and immunohistochemical staining revealed focal high-grade urothelial carcinoma. **(D)** Postoperative pathological diagnosis revealed that the morphology of the bladder cancer was consistent with high-grade, invasive urothelial carcinoma with squamous metaplasia, infiltrating the whole bladder wall, and vascular invasion with a tumor thrombus. **(E, F)** On October 23, 2023, urinary tract-enhanced CT revealed no abnormal enhancement in the pelvic wall or lower abdomen, no thickening or abnormal enhancement was observed in the rectum wall, and no hydronephrosis or hydroureter was observed in either the kidneys or ureters. **(G, H)** On July 04, 2024, urinary tract-enhanced CT revealed that, compared with the previous examination, the imaging changes were not significant.

An 85-year-old man was admitted to the Second Affiliated Hospital, Army Medical University, on July 17, 2023, with the chief complaints of frequency, urgency, and odynuria for 6 years and 10 months, occasionally accompanied by hematuria. The patient experienced urinary incontinence and nocturia but did not have other clinical symptoms or signs. The patient underwent transurethral resection of the prostate (TURP) at the local hospital 10 months prior, and the postoperative pathological diagnosis was unknown. The above symptoms did not improve significantly. Urinary tract computed tomography (CT) on July 04, 2023, at the Second Affiliated Hospital, Army Medical University revealed that the right sidewall of the bladder was not uniformly thickened, a mass with soft tissue density was visible, the size was approximately 5.5*5.2 cm, and the pelvic lymph nodes were enlarged (**Figures 1A, B**). The enlarged prostate was palpated via digital rectal examination (DRE). On July 06, 2023, the patient underwent cystoscopy plus biopsy. A mass in the right wall of the bladder was observed during the operation, the size of the mass was approximately 5.5*5.2 cm, its base was broad, and the surface was bleeding. Postoperative pathological diagnosis combined with imaging, morphology, and immunohistochemical staining revealed focal high-grade urothelial carcinoma (**Figure 1C**). The patient had a history of type 2 diabetes, chronic bronchitis and emphysema. The family history and physical examination of the patient were not exceptional.

There were no obvious abnormalities on chest radiography or abdominal ultrasound. The patient was eventually diagnosed with bladder cancer (cT1G3NxM0). Considering the large size of the bladder tumor, the patient was advised to undergo platinum-based neoadjuvant chemotherapy before RC. Because of concerns about the adverse events of platinum-based chemotherapy, the patient requested RC plus PLND directly. The patient hoped to have a comparatively better quality of life after RC and strongly requested ileal conduit urinary diversion. However, on July 21, 2023, during the operation, the bladder cancer had invaded the small intestine, colon and right pelvic sidewall. After communication with the authorized relatives of the patient, it was decided to perform a partial enterectomy plus simultaneous ureterostomy. Owing to the risk of enterostomy after partial colectomy, which may further affect the quality of life of the patient and increase the risk of surgery, the authorized relatives of the patient decided not to undergo partial colectomy. Finally, we finished radical cystectomy, simultaneous ureterostomy, pelvic lymph node dissection and partial enterectomy.

**Figure 2:**
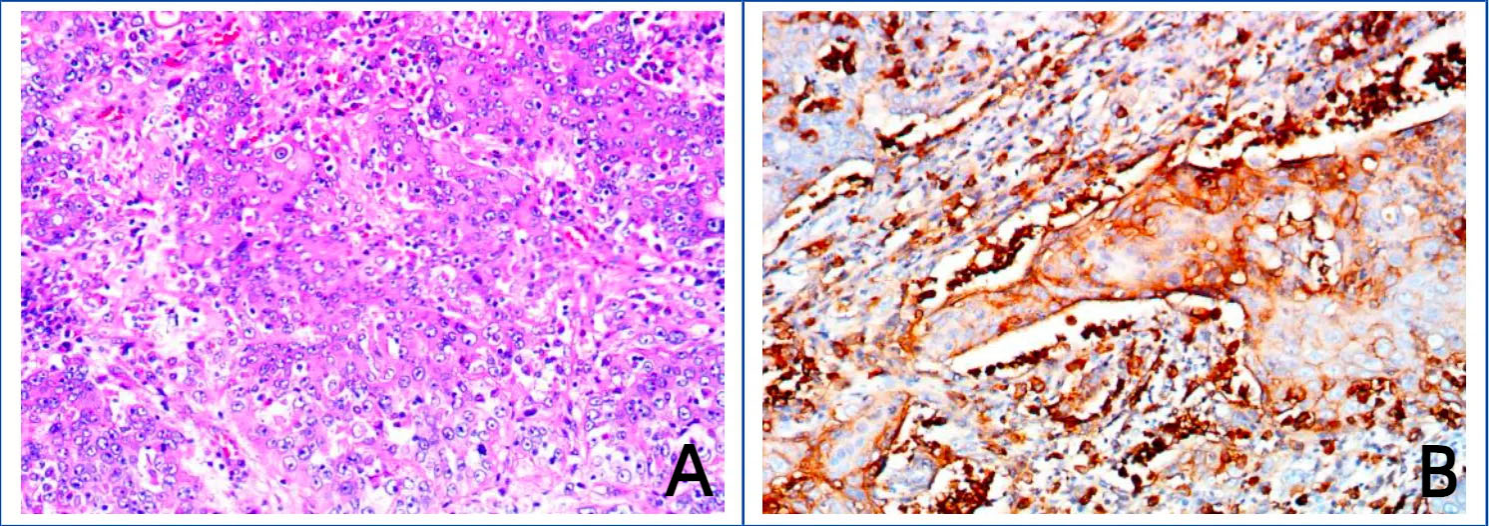
Images of hematoxylin–eosin (HE) staining of bladder cancer tissue and immunohistochemical staining of PD-L1. **(A)** Hematoxylin–eosin (HE) staining of bladder cancer tissue. **(B)** Immunohistochemical staining of PD-L1.

The patient was given nutritional support after surgery. The time to first flatus of the patient was the 3rd day after surgery, the time to first bowel movement was the 5th day after surgery, the time to regular diet was the 7th day after surgery, and the length of hospital stay was 10 days. Postoperative pathological diagnosis revealed that the morphology of the bladder cancer was consistent with high-grade, invasive urothelial carcinoma with squamous metaplasia infiltrating the whole bladder wall, and vascular invasion with a tumor thrombus could be observed (**Figure 1D**). No cancer was found in the prostate, bilateral seminal vesicle, vas deferens or ureter of the margins after resection. Bladder carcinoma metastasized to the small intestine, right pelvic sidewall and pelvic lymph node (2/10). Following resection, no cancer was found in the small intestine of the bilateral margins. Next-generation sequencing (NGS) results revealed missense mutations in CDKN2A (p.A68Rfs*52, mutation level: 46.12%) and TP53 (p.V143A, mutation level: 25.13%) (**Table 1**). Hematoxylin–eosin (HE) staining revealed bladder cancer (**Figure 2A**). Immunohistochemical staining of PD-L1 revealed that the tumor proportion score (TPS) was 20%, and the combined positive score (CPS) was 50% (**Figure 2B**). The patient was eventually diagnosed with BC (pT4bN2M1b).

**Table 1:** Results of next-generation sequencing (NGS) of the patient tumor tissue.

Gene	Position	Base alteration	Amino acid alteration	Mutation abundance
CDKN2A	Exon 2	c.200dup	p.A68Rfs*52	46.12%
TP53	Exon 5	c.428T>C	p.V143	25.13%
TET2	Exon 11	c.5618T>C	p.I1873T	1.9%
ABRAXAS1	Exon 5	c.307C>T	p.R103C	1.06%

From September 04 to November 23, 2023, the patient accepted and started 4 cycles of gemcitabine (1000 mg/m2, Days 1 and 8, intravenously (i.v.), once every 21 days) combined with cisplatin (70 mg/m2, Day 2, intravenously (i.v.), once every 21 days) plus toripalimab (240 mg, Day 1, intravenously (i.v.), once every 21 days). Urinary system-enhanced CT on October 23, 2023 revealed no abnormal enhancement in the pelvic wall or lower abdomen, no thickening or abnormal enhancement was observed in the rectum wall, and no hydronephrosis or hydroureter was observed in either the kidneys or ureters (**Figures 1E, F**). Both liver and renal function were within the normal range. A chest CT on October 23, 2023 revealed several small solid pulmonary nodules in both lungs that were approximately 2–3 mm in diameter. After 4 cycles of chemotherapy and immunotherapy, the patient continued a 21-day cycle of toripalimab as maintenance immunotherapy. Urinary system-enhanced CT and chest CT on July 04, 2024 revealed that, compared with the previous examination, the imaging changes were not significant (**Figures 1G, H**).

## Discussion

In recent years, the development of ICIs has revolutionized cancer treatments. Although immunotherapies affect the immune system and thus cause immune-mediated adverse events (imAEs), these imAEs are generally manageable with early and appropriate interventions (14). Mao J. reported two cases of recurrent MIBC. One patient received neoadjuvant chemotherapy (NAC) plus maximal transurethral resection of bladder tumor (TURBT) and postoperative immunotherapy. The other patient was given maximal TURBT combined with postoperative adjuvant intravesical chemotherapy plus immunotherapy (15). Postoperative immunotherapy should be considered a potential alternative treatment option for recurrent MIBC patients. In a retrospective study regarding bladder cancer treatments, 31 patients with locally advanced or metastatic bladder cancer received immunotherapy plus gemcitabine combined with cisplatinum (GC) or GC alone, respectively. The results suggested that patients treated with immunotherapy plus GCs achieved better antitumor effects and safety than those treated with GCs alone (16).

The patient with metastatic MIBC received 4 cycles of toripalimab plus GC chemotherapy for adjuvant therapy after RC at the Second Affiliated Hospital, Army Medical University. Imaging data revealed no evidence of remnant tumor cells in the pelvic cavity, and the metastases to the colon disappeared. The patient subsequently received maintenance therapy with toripalimab for 1 year. To date, the patient has relatively good renal function and quality of life without MIBC recurrence or metastasis. PD-1 is expressed in activated T lymphocytes, adjusts effector T-cell function and suppresses the immune responses of T cells (17). As PD-1 combines with its ligand, PD-L1, T-cell activity decreases, and exhaustion occurs. It is now thought that this is one of the mechanisms to prevent autoimmunity (18). In MIBC patients, some studies have shown that, compared with that in normal bladder tissue, PD-1/PD-L1 expression is higher in tumor samples (19, 20). Moreover, the PD-L1 expression level in tumor tissue is related to increased tumor grade and stage, with high expression in Bacillus Calmette Guerin (BCG)-unresponsive patients (21). Standard treatment for metastatic MIBC has long been limited to platinum-based chemotherapy. For some cisplatinum-intolerant patients, the approval of first-line immunotherapy relies on the PD-L1 expression level, namely, the TPS and CPS. Employing TPS and CPS immunohistochemical staining may maximize the status of first-line ICIs to treat MIBC patients who are unfit for cisplatinum-based chemotherapy (22).

Recently, the development of NGS has improved the genomic characterization of bladder cancer, which may improve the understanding of the genetic underpinnings of disease and drug response for clinicians and allow further customization of treatments and prediction of individualized therapeutic responses. Given the unsatisfactory objective response rate (ORR) of ICIs as first-line therapies in UC patients, identifying biological indicators for predicting the efficacy of ICIs via whole-genome sequencing is very important for further clinical application of ICIs. The cyclin-dependent kinase inhibitor 2A (CDKN2A) gene, also known as the P16 gene, encodes multiple tumor suppressor 1 (MTS1) (23). Compared with that in normal tissue, the expression level of CDKN2A is greater in tumor tissue, which can be a biomarker and reflect the prognosis of cancers. In assays of immune cell infiltration, high CDKN2A expression in tumor tissue was obviously positively related to increased numbers of activated immune cells, which suggested that CDKN2A may play a key role in tumor immunity (24). CDKN2A has great potential as a target for immunotherapy.

DNA damage response and repair (DDR) defects play a vital role in the occurrence, development, therapeutic response to immunotherapy, and prognosis of bladder cancer. An increasing number of DDR-related genes have been studied. Teo and colleagues analyzed 34 DDR genes in several pathways. In patients with metastatic UC receiving nivolumab (anti-PD-1 antibody) or atezolizumab (anti-PD-L1 antibody), harmful DDR alterations are related to longer OS (25). Biallelic mutations in genes of DDR pathways, such as tumor protein P53 (TP53), are also significantly related to increased tumor immunogenicity (26). Based on the data from this case and the above immunotherapy cohort, we hypothesized that high PD-L1 expression as well as CDKN2A and TP53 mutations could predict immunotherapy efficacy and prognosis in patients with BC.

Our case suggested that RC plus PLND combined with adjuvant therapy (GC chemotherapy plus toripalimab) may be an encouraging treatment option for patients with metastatic MIBC. In addition, a high TPS of PD-L1 expression plus CDKN2A and TP53 mutation levels may predict the efficacy of immunotherapy and patient prognosis.

## Data Availability

The original contributions presented in the study are included in the article/supplementary material. Further inquiries can be directed to the corresponding authors.
